# ‘The way to obtain freedom and equality’: Experiences and needs of Thai adolescent mothers in terms of the use smartphone applications for breastfeeding support

**DOI:** 10.1371/journal.pone.0300041

**Published:** 2024-04-01

**Authors:** Sasitara Nuampa, Sudhathai Sirithepmontree, Metpapha Sudphet, Crystal L. Patil

**Affiliations:** 1 Department of Obstetrics and Gynecological Nursing, Faculty of Nursing, Mahidol University, Bangkok, Thailand; 2 Department of Obstetrics and Gynaecology, Siriraj Hospital, Mahidol University, Bangkok, Thailand; 3 Department of Health Behavior and Biological Sciences, School of Nursing, University of Michigan, Ann Arbor, Michigan, United States of America; Christiana Care/University of Delaware, UNITED STATES

## Abstract

**Background:**

Breastfeeding brings about a positive impact on both child and maternal health in the short and long terms. However, adolescent mothers have a lower breastfeeding initiation rate and a shorter breastfeeding duration than adult mothers. Although mobile applications have been found to be the most widely used platform for breastfeeding support, there is still a lack of design specific for adolescents.

**Aim:**

To explore the experiences, perspectives and needs of adolescent mothers and professional nurses using smartphone applications for breastfeeding support and the influence of this technology on healthcare in Thailand.

**Methods:**

This formative qualitative research of the ‘Development of smartphone application for promoting breastfeeding and learning of infant responsiveness for Thai teenage pregnant women’ study intends to design a smartphone application and develop an implementation plan. A purposive sample was used to elicit experiences from adolescent mothers and nurses. Data were collected from 48 adolescent mothers through in-depth interviews with six focus group discussions and 12 nurses, following the data saturation principle. Thematic analysis was conducted, and potential factors and needs were mapped to the capability, opportunity, and motivation model of behaviour change (COM-B).

**Results:**

Most adolescent mothers perceived the usefulness of smartphone apps as breastfeeding support resources. The qualitative findings of adolescent mothers were grouped into the following three themes: a friendly breastfeeding tool; allows them to manage their breastfeeding activities; and enhances the accessibility and equality of breastfeeding support. The professional nurses perceived the benefits of using smartphone applications in their work, which included the following three themes: reducing workload and making their work easier; preparation is always better; and increasing the standards of breastfeeding support.

**Conclusion:**

Adolescent mothers and professional nurses showed favourable attitudes toward smartphone apps for breastfeeding support. These smartphone apps should be tailored to these groups to achieve optimal BF outcomes.

## Introduction

Recent epidemiological and biological findings from the past decade expand on the known positive impact of breastfeeding (BF) on child and maternal health in both short and long terms [1]. The World Health Organization recommends exclusive breastfeeding (EBF) for the first 6 months of an infant’s life and combined feeding for the first 2 years; this recommendation is reflected in the global public health guidance [2]. Additionally, the 6-month EBF was set as a 2025 global target to increase by at least 50% [3]. Nevertheless, the BF rates worldwide have not reached optimal levels; in particular, the East Asia and Pacific region has the second lowest rate of EBF for the first 6 months of an infant’s life at 31% [4]. In Thailand, the EBF rate decreased from 23.1% in 2016 to 14% in 2019, which was far below the global target [5].

In some studies, adolescent mothers have a lower prevalence of BF initiation and a shorter BF duration than adult mothers [6, 7]. Adolescent mothers who were unable to continue EBF or wean their children explained their psychological difficulties as a repeating mistake [8]. Contrarily, successful EBF can provide young mothers with a sense of accomplishment and improved their self-esteem in their maternal role [9, 10]. Breast milk is the most nutritious food for babies; hence, BF considered is a morally superior act that demonstrates good motherhood [11, 12]. Furthermore, BF could improve maternal perception by increasing the level of maturity and responsibility among adolescent mothers [12]. BF success in this group appeared to be strongly linked to both physical and psychological benefits.

The previous systematic evidence presented that only a combination of education and counselling provided by a lactation consultant–peer counsellor team significantly improved both BF initiation and duration among adolescent mothers [13]. According to a Brazilian study, counselling interventions and adding BF information could increase the rate of EBF at 2 months and that cohabiting with grandmothers did not affect the EBF duration [14]. Ylmaz et al. [15] also found that adolescent mothers receiving BF education and encouragement had longer BF duration. Even though adolescent mothers have a high risk for BF complications, BF education and counselling have positive effects and were able to prolong the BF practices in this vulnerable group.

The rate of technology use has been increasing; in fact, most mothers depend on technology-based BF approaches [16]. The pooled results of the systematic review indicated that studies using electronic-based interventions had a higher effect on BF than provider-based interventions [17]. In the recent evidence that technology supports BF, most interventions were used in the postpartum period, which frequently includes web research, text messaging and mobile applications. Communication tools or counselling for solutions appear to be more effective for BF mothers as compared to game interventions [18–20]. Despite the fact that mobile applications are the most commonly employed platform to support BF, including location-mapping [21], BF support [22], empowering mothers [23], tracking BF [24] and BF information [25], there is still a lack of design to support BF among adolescent mothers. Moreover, some gaps were identified in the quality of information, acceptability, and effectiveness of mobile applications [26]. Thus, it is critical to tailor BF education and consultation for adolescent mothers using a high-tech platform and a mobile application. To improve the acceptability and maintain the effectiveness of education and counselling support, the BF information and digital technology used should be designed for the needs of the target group. Therefore, this formative qualitative research aimed to investigate adolescent mothers’ experiences, perspectives, and needs for BF support using smartphone applications while also exploring professional nurses’ perceptions of the use of technology for BF support and related works.

## Materials and methods

### Design

This formative qualitative study of the major project ‘Development of Smartphone Application for Promoting BF and Learning of Infant Responsiveness for Thai Teenage Pregnant Women’ used focus groups and in-depth interviews. The qualitative research methodology allows researchers to capture the way individuals react, understand, and think about the questions through the dynamics of the situation and the perspective of the individual who is living it [27]. This study conforms to the Standards for Reporting Qualitative Research (SRQR) [28]. Ethical approval was provided by the Multi-Faculty Cooperative Institutional Review Board (MU-MOU CoA No. IRB-NS2022/650.1601). Written electronic informed consent was obtained before starting the focus groups and interviews with audio recording.

### Setting

The study was conducted at a tertiary hospital in Bangkok, the largest in Thailand, with patients of various characteristics. The Statistical Hospital Report in 2021 showed that the number of in-patients in the Obstetric and Gynecological Department was approximately 15,000 per year. The hospital served 3,000 pregnant women per year, with 10% of them being adolescent mothers. Regarding the standard of service, the antenatal care unit has a teenage pregnancy clinic (Pink-Star Clinic), which manages the continuing-care system through midwifery.

### Sample

This study had two groups of participants, consisting of adolescent mothers and obstetric and paediatric nurses. The participants were a purposive sample of adolescent mothers who gave birth during the first 6 months and professional nurses who were working in the obstetric and paediatric nursing departments. For the adolescent mothers, the inclusion criteria were as follows: age, 15–19 years; BF experience of at least 1 week; experienced using mobile applications for health; and cohabitating with an infant. They were excluded from the study if they had a psychological illness. For nurses, the inclusion criteria were as follows: work involving BF promotion and support and adolescent care for at least 1 year. The principle of saturation of new emerging themes was used to determine when to stop with data collection. This was achieved by the eligible participants, which included 48 adolescent mothers and 12 nurses.

### Data collection

The data were collected from April to October 2022. In adolescent mothers, we first identified the potential participants from the postpartum medical records and then contacted eligible mothers via telephone to explain the study and invite them to participate without coercion. The mothers who agreed to participate signed an electronic informed consent form and set an available date and time to conduct an online focus group via Zoom meeting. The adolescent mothers were assigned into six focus group discussions (FGDs), with eight participants allocated in each group depending on the postpartum duration, from 1 to 6 months after childbirth. Altogether, 48 participants provided their experiences and perceptions related to technology-based BF support, in particular smartphone applications. The researcher sent a Google Form link via the LINE application prior to the start of the FGDs to gather the participants’ demographic, pregnancy and childbirth data, BF information and health application user data, which comprised a total of 31 items.

For the professional nurses, we recruited potential participants through a poster announcement in the obstetric and paediatric nursing offices. This study included 2–3 nurses from each setting where they provided BF and adolescent mothers’ support, including the antenatal care unit, delivery unit, postpartum unit, lactation clinic, and well-baby clinic, to investigate their perceptions of technology-based BF support as well as their roles. The eligible participants who were willing to share their opinions signed an informed consent form before undergoing in-depth, semi-structured interviews. We conducted 12 key informant in-depth interviews (IDIs) with the participants, including eight obstetric and four paediatric nurses.

The FGDs were used 60–90 and 45–60 minutes for IDIs. All FGDs and IDIs were conducted by expert qualitative researchers with >5 years of experience and were health care providers. All enrolled individuals received reimbursements as gratitude for participating in the study.

### Data collection instruments

The focus group and interview guides were developed by researchers based on a literature review [25, 29, 30]. The focus group guides included 14 open-ended questions designed to elicit participants’ BF technology experiences, perspectives, and technology needs for supporting BF in adolescent mothers, such as ‘Could you tell me about a technology BF support you have ever used?’, ‘What made you think you needed a smartphone application to help with BF support?’ and ‘What are the benefits of using smartphone applications to promote and support BF?’. The 13-item interview guide for key professional nurses included open-ended questions to understand their perspectives on BF support technology and their roles on their work, such as ‘What do you think about the BF support technology for adolescent mothers?’, ‘If you have the opportunity to participate in the development and use of processes, what should be added to this instrument?’ and ‘How has the smartphone application for BF support among adolescent mothers influenced your daily work?’.

For the other measurements, the adolescent mothers were distributed three online self-administered questionnaires developed by researchers, including the Demographic, Pregnancy, and Childbirth Information (17-item questions, e.g. age, marital status, educational status, occupation, income, pregnancy plan, gestational age at delivery, and infant age), BF Information (6-item questions, e.g. BF intention, BF status, and BF problems) and Mobile Health Application User Information (8-item questions, e.g. internet use, health and BF application use, and satisfaction). EBF in this study was defined as providing only breast milk from the mother or wet nurse for the first 6 months without other solids or liquids, except for drops or syrups consisting of vitamins, minerals, supplements, or medicines. BF duration was defined as continuous BF from birth, including feeding with water, liquid, food, or formula milk. Participants were asked how old their infant was when they stopped BF to determine the BF duration [31]. For professional nurses, a 6-item, closed-ended questionnaire of The Personal and Work Information was used. The questionnaires, focus groups and interview guides were evaluated for content by three experts.

### Data analysis

Each FGD and IDI was audio recorded and later transcribed. Transcripts in Thai version were analysed by three Thai expert qualitative research team members (SN, SS, and MS), who separated the thick and individual immersing data and through a meeting to discuss the emerging key themes. This study used open coding and thematic analysis based on the themes that emerged from the participants’ narratives. Open coding of transcripts using manual analysis allowed us to obtain multiple codes that capture experiences, perceptions, and needs related to high-technology BF support. Additional themes emerged through the process of combining multiple codes.

To ensure trustworthiness, confirmability was conducted through an audit trail by using audio-recordings and immediately recording them in the field notes. Moreover, rigorous thematic analysis and peer debriefing with the research team were conducted to confirm the credibility. To preserve the original meaning, data analysis was undertaken in Thai, and excerpts from transcripts in this article were translated by a bilingual Thai-English translator who is a part of the research team [32, 33].

These research findings were then conceptualised for implementation and mapped to the capability, opportunity, and motivation model of behaviour change (COM-B) [34]. The COM-B theories state that for a desired behaviour to occur (e.g. BF practices), individuals must have the capability, opportunity, and motivation to enact the behaviour. Capability refers to an attribute of a person such as attention, decision-making, knowledge, and skills. Opportunity refers to a system of attributes that influences behaviour and includes both the physical (e.g. access to supplies and resources, staffing and infrastructure) and social (e.g. team support, practice norms and social and professional identities) aspects. Motivation refers to an aggregate of mental processes that energise and direct behaviour [34, 35]. The COM-B model has been widely used in research to improve implementation and change clinical practice. By identifying factors that may affect implementation, teams can then design implementation strategies to address these factors.

## Results

The adolescent mothers’ average age was 18 years (standard deviation [SD] = 1.27). Even though, most adolescent mothers reported having sufficient family income (n = 39, 62.5%), the average family income was 541.07 USD per month (SD = 638.45). For the pregnancy data, nearly half of them had their first antenatal care (ANC) visit late, with a mean of 14.66 weeks (SD = 7.69); 12.5% (n = 6) did not visit an ANC clinic. Table 1 presents the characteristics and pregnancy information.

**Table 1:** Characteristics and pregnancy information among adolescent mothers (n = 48).

Characteristics		n (%)
**Maternal age (year)**	15–17	15 (31.3)
	18–19	33 (68.7)
**Marital status**	Cohabitation	23 (47.9)
	Married	21 (43.8)
	Separated	4 (8.3)
**Occupation**	Housewife	23 (47.9)
	Business	12 (25.0)
	Full-time employee	6 (12.5)
	Student	6 (12.5)
	Part-time employee	1 (2.1)
**Income status**	Sufficient income	30 (62.5)
	Insufficient income	18 (37.5)
**Family character**	Nuclear family	26 (54.2)
	Extended family	22 (45.8)
**Pregnancy Plan**	Intended	8 (16.7)
	Unintended	40 (83.3)
**Gestational age (week)**	<37	2 (4.2)
	37–40	42 (87.4)
	>40	4 (8.4)
**First ANC (week)**	1–12	25 (52.1)
	13–28	22 (45.8)
	>28	1 (2.1)
**Mode of delivery**	Vaginal delivery	31 (64.6)
	Caesarean section	17 (35.4)

Regarding BF, almost all adolescent mothers (n = 46, 95.8%) intended to breastfeed their child, with 62.4% (n = 30) of them intending to do long-term BF of <6 months. In fact, half of them could breastfeed within the first 3 months, with the average BF duration being 3.5 months (SD = 1.72). Moreover, the average EBF duration was 2.12 months (SD = 1.35), with 79.1% (n = 38) of them EBF between 1 and 3 months. In this study, the key barriers to EBF were the beliefs that adding water prevents jaundice, thirst, and hiccups in infants; perceived insufficiency in milk supply; and BF difficulties (positions and attachment). We discovered that 14 infants received water before the age of 6 months, with nearly half of them receiving it within the first month (n = 7, 49.9%). Twenty-six infants received early formula milk, and almost all of them started it within the first 2 months of life (n = 19, 72.9%). Finally, only seven infants were introduced to early foods at 6 months of life, and they were fed their first foods at 4 (57.1%) and 2 (28.6%) months. The mean age for introducing formula milk, water and semi-solid food was 2.03 (SD = 1.42), 2.37 (SD = 1.79) and 3.85 (SD = 0.69) months, respectively.

All the adolescent mothers in this study used the internet daily. More than half of adolescent mothers used the BF app, with a feature usage rate of 3.6 (SD 2.7). The internet and mobile application experiences and perceptions are shown in Table 2.

**Table 2:** The users’ experiences on internet and smartphone applications (N = 48).

Users’ experiences		n (%)
**Application download**	Yes, free only	30 (62.5)
	Yes, both free and paid	10 (20.8)
	No	8 (16.7)
**Numbers of favor health application**	No one	31 (64.6)
	1–2 applications	17 (35.4)
**Needs for breastfeeding application**	Extremely need	17 (35.4)
	Very need	9 (18.8)
	Moderate need	12 (25.0)
	Minor need	8 (16.7)
	None	2 (4.2)
**Breastfeeding application’s use**	Use	28 (58.3)
	No use	20 (41.7)
**Application feature’s use (n = 154)**	Feeding record	20 (12.9)
	Pumping record	13 (8.4)
	Diaper changing record	18 (11.7)
	Infant sleep record	18 (11.7)
	Diary	17 (11.0)
	Searching for feeding room	6 (3.9)
	Breastfeeding information	19 (12.4)
	Solving breastfeeding problem information	24 (15.6)
	Expert consultation	19 (12.4)
**Perception for breastfeeding application (n = 28)**	Not enjoyable	3 (10.7)
	Not attractive	1 (3.7)
	Need to be improved	2 (7.1)
	Be interesting	20 (71.4)
	Be enjoyable	2 (7.1)
**Satisfaction level for breastfeeding application (n = 28)**	Extremely satisfaction	6 (21.4)
	Very satisfaction	5 (17.7)
	Moderate satisfaction	12 (42.9)
	Minor satisfaction	4 (14.4)
	unsatisfaction	1 (3.6)

Moreover, adolescent mothers purposed their needs for BF support through smartphone applications, both BF contents and experiences involving maternal and child aspects. They preferred to start using the application during pregnancy to prepare for and prevent several BF problems after childbirth. In addition, they recommended essential features for an ideal BF application, as presented in Table 3.

**Table 3:** Adolescent mothers’ needs for breastfeeding support from smartphone applications.

Smartphone applications’ needs		n (%)
**Breastfeeding contents (n = 141)**	The importance of a 6-month EBF	21 (14.9)
	Growth and development for breastfeeding infants	20 (14.2)
	Solving common problems for breastfeeding (crack nipple, oversupply milk, insufficient milk)	19 (13.5)
	Techniques for the correct positions	18 (12.8)
	Nutrition for lactating mothers	15 (10.6)
	Techniques for pumping	14 (9.9)
	Maternal stress and coping during breastfeeding	12 (8.5)
	Assessment of breast milk supply	9 (6.4)
	Techniques for increasing breast milk supply	7 (4.9)
	Preparing milk supplies before returning to school or work	6 (4.3)
**Application features (n = 179)**	Enjoy and engage	42 (23.5)
	Decision-aid tool	40 (22.3)
	Interactive functions	36 (20.1)
	Expert chat for counseling	29 (16.2)
	Peer chat	17 (9.5)
	Motivation activity	15 (8.4)

For professional nurses, the average age was 38.5 years (SD = 10.05). The average work experience was 16.08 years (SD = 10.53; range: 3–36 years). Regarding BF support experiences, they had 9.67 years of experience (SD = 7.04; range 2–22 years). The perception of engagement in BF support is shown in Table 4.

**Table 4:** The work experiences of obstetric and paediatric nurses (n = 12).

Age (year)	Working Unit	Working experience (year)	BF experience (year)	Engagement perception
26	ANC	3	2	Moderate
44	ANC	22	22	Moderate
28	LR	6	2	High
40	LR	17	7	High
48	LR	28	15	High
27	PP	4	4	Moderate
42	PP	18	18	High
58	PP	36	10	High
36	LC	13	5	High
46	LC	24	12	High
27	WB	4	2	High
40	WB	18	17	High

Note: ANC = Antenatal Care Unit; LR = Labor Unit; PP = Postpartum Unit; LC = Lactation Clinic; WB = Well-baby Clinic

### Qualitative findings for adolescent mothers’ perspectives

Six FGDs disclosed their experiences, perspectives and needs for BF support via smartphone applications, which consisted of the following three main themes: a friendly BF tool; allow them to manage their BF practice; and enhances accessibility and equality for BF support.

### A friendly BF tool

Almost all adolescent mothers perceived smartphone applications as modern tools for BF mothers. Especially adolescent mothers mentioned friendly characters for users such as freedom, convenience, and safety which allowed them to search for information at any time and from anywhere. Furthermore, these tools were effective in preventing miscommunication, a social skill deficit among adolescents. There were two subthemes, including 1) freedom and less time-consuming to obtain BF information and 2) avoidance of misunderstandings in conversation.

#### Freedom and less time-consuming to obtain BF information

All adolescent mothers accepted that some problems or doubts about BF problems or concerns were uncomfortable to raise directly with family members or healthcare providers. Moreover, self-searching via an online platform helped them to avoid negative judgements. This platform served their needs at anytime and anywhere. In addition, the application gathered all relevant information to reduce their searching times:

‘*I will search on the internet about what I can eat besides ginger ale*. *I can’t eat every day*. *Can I eat something else*? *I want to drink cold water*. *If I ask Grandma*, *she’ll tell me that I can’t eat like this’*. *(1-month postpartum group)*

“I think it’s (the mobile application) better than searching for each website that takes days and is difficult to choose because it was both believable and unbelievable. I had a hard time making a decision. If there was a website or an app that included everything about breastfeeding and raising babies, it would be convenient for me to open and read it without wasting my time’. (3-month postpartum group)

#### Avoidance of misunderstandings in conversation

Several adolescent mothers stated that they lacked the confidence to communicate directly with healthcare providers, especially when confronted with difficult situations involving BF issues or infant behaviours. They prefer to type their questions instead. Importantly, face-to-face conversations generated feelings of pressure and coercion.

‘*I’m afraid I’ll confuse the lactation nurse when I talk with her*. *I’d rather type and then let her answer my queries afterwards; it’ll be OK*. *If I call her personally*, *I’m usually nervous and forget important details*, *such as when my son was sick*. *My son may be in danger if I do not mention certain symptoms’*. *(1-month postpartum group)*

### Allow them to manage their BF practice

Most adolescent mothers described their experiences and needs when they used an online platform and BF application that facilitated them to manage their times, search for answers and solve some basic problems. There were two subthemes, including 1) facilitating the review of BF knowledge and 2) a tool that supports decision-making among BF mothers.

#### Facilitating the review of BF knowledge

All mothers agreed that a smartphone application could collect comprehensive BF information that facilitated their review of BF information every time. They described their experiences regarding overcrowding in hospitals and how healthcare providers had a limited time when providing BF information:

‘*Suppose I forget*, *I can go back and read the information again*. *If I call the nurse and then forget the next day*, *because I tend to forget many things*, *especially those that are confusing*, *it might be annoying when I call the nurse again’*. *(4-month postpartum group)*

#### A tool that supports decision-making tool among BF mothers

Some adolescent mothers agreed that smartphone applications could help them make decisions and manage their BF and baby care activities. Adolescent mothers described their experiences with BF problems and babies’ illnesses. They expressed their need for practical information that could help them assess and decide for themselves and showed an alert notification:

‘*When my baby gets sick*, *such as a stomachache*, *I would search for basic information on how to manage the illness before deciding to go to the hospital*. *I don’t want to go to the hospital right away because I’m afraid of acquiring an infection or any disease’*. *(3-month postpartum group)*

‘*It reminds me to breastfeed every2 hours*. *It is important for newborns to feed on time*. *If I do not breastfeed on time*, *the baby will be malnourished and I will have breast pain as well*. *This will help moms a lot’*. *(6-month postpartum group)*

### Enhance accessibility and equality for BF support

Adolescent mothers in this study emphasised the value of smartphone applications, as these allowed them to consult with experts directly. Several mothers described difficulties in getting to the hospital, such as a lack of family support and insufficient funds for transportation. As a result, they agreed that a smartphone application with provider services would help them resolve BF issues and make professional services more accessible. This theme was divided into two subthemes: 1) easy access to BF support and 2) saving time and money.

#### Easy access to BF support

Most of the adolescent mothers in this study reported that they had a hard time availing hospital care services because they did not have support from their families. Half of them came from nuclear families, and their spouse had to work full-time. The smartphone application will allow them to consult with BF experts without going to the hospital:

‘*For convenience*, *I lived far from the hospital*, *and my husband could not take me there because of his full-time work*. *So*, *I’d rather choose to talk to the experts via the app*. *If I go to the hospital*, *it may take around 3–4 hours to talk with a doctor*. *Moreover*, *I have to wait for a long time within the crowd of patients’*. *(5-month postpartum group)*

#### Saving time and money

Almost all adolescent mothers agreed that the BF smartphone application could provide effective support. This type of assistance reduced the transportation costs and saved time, especially for those with limited income:

‘*The advantage is that I don’t have to go to the hospital; that is*, *there is no need to pay for the transportation*. *I have to take 500 baths (16 USD) from home*. *I’m not working*. *I feel sorry for my husband’*. *(1-month postpartum group)*

‘*If I go to the hospital*, *I have to spend a lot of money for travel*, *food and medicine expenses*. *Sometimes*, *I don’t have enough… I will also take a leave from work*. *If there is an application that I can use*, *I want to try to solve the problem first by myself before going to the hospital’*. *(5-month postpartum group)*

### Qualitative findings for obstetric and paediatric nurses’ perception

All of the nurses who work in areas involving BF promotion and support, as well as those involved in adolescent mothers’ care, disclosed some adolescent mothers’ positive viewpoints about the use of a smartphone application. They mentioned that it has the potential to increase the access and reduce costs for this vulnerable group. Furthermore, the nurses found benefits of the application on their work, which included three themes: reduced their workload and made their work easier; preparation is always better; and increased the standards of BF support.

### Reduced the nurses’ workload and make their work easier

Almost all of the nurses in this study expressed a favourable opinion of the use of smartphone applications for BF support, claiming that they would reduce their workload and make their jobs easier. They mentioned that if adolescent mothers have the opportunity to self-study using credible information available in the smartphone applications, they may have a foundation of knowledge. The benefits will include simplification of the work of nurses in terms of communicating BF information and an increase in the exchange of information. This will reduce the workload in terms of solving time-consuming problems. However, several nurses disclose that their current workload limits them from thoroughly informing teen mothers about BF:

‘*If the mothers have a tool that provides them knowledge*, *this may help reduce my workload*. *Because if they have reliable information*, *they will learn to take care of themselves and prevent the development of some common breastfeeding problems*. *They may also be confident enough to speak with us’*. *(Postpartum nurse*, *working for 18 years)*

‘*The app will help make our work easier*. *It will shorten the work process because mothers will be able to properly manage their breastfeeding practices; thus*, *problems will be reduced*. *Especially*, *if mothers breastfeed their child appropriately*, *the incidence rate of neonatal jaundice will decrease*. *Thus*, *my workload will be decreased as well’*. *(Well-baby clinic nurse*, *working for 18 years)*

### Preparation is always better

Most nurses had a reach a consensus on the benefits of technology or smartphone applications for promoting and supporting BF education as a good preparation for adolescent mothers, particularly early in their pregnancies. Adolescent mothers would be able to understand the benefits of BF for themselves and their infants; this will create BF intention. In addition, teenage mothers will also be educated on possible future problems; hence, they can come up with a preventative plan of time. A well-prepared intervention plan will contribute to good BF outcomes while also facilitating easy communication and supporting satisfaction on BF service:

‘*The advantage will be evident in the postpartum unit*, *as they will have enough knowledge to start and continue breastfeeding*. *Most importantly*, *they will understand why I have to assist them in breastfeeding or wake them up every 2–3 hours to breastfeed their infant*. *I think it’s always better to be prepared’*. *(Antenatal clinic nurse*, *working for 22 years)*

### Increase the standards of BF support

A minority of nurses in the study, who had less than 5 years of experience with BF support, expressed that the use of the smartphone application guided them in providing information to adolescent mothers. They felt that the quality of BF support may depend on the nurses’ work experience; thus, standardising BF information will ensure equal care for all mothers:

‘*For me*, *when I compared the experiences I had supporting adolescent mothers and their breastfeeding problems*, *I had fewer experiences as compared to those of the senior nurses*. *If breastfeeding information in a smartphone app is standardised*, *it will be an educational tool for nurses as well*. *For now*, *I observed that we have different suggestions for moms’*. *(Antenatal clinic nurse*, *working for 3 years)*

‘*I think having this application will help create a link among the hospital units*. *For example*, *the time it takes for the delivery and postpartum nurses to understand the problem or status of a teenage mother will be shortened*, *as they have read the previous information from the antenatal unit; hence*, *they will provide the appropriate care more quickly*. *Specifically*, *every nurse will be aware of these data*, *guiding their interventions’*. *(Postpartum nurse*, *working for 4 years)*

### Understanding the stakeholders’ perceptions affecting implementation using the COM-B model

**Fig 1:**
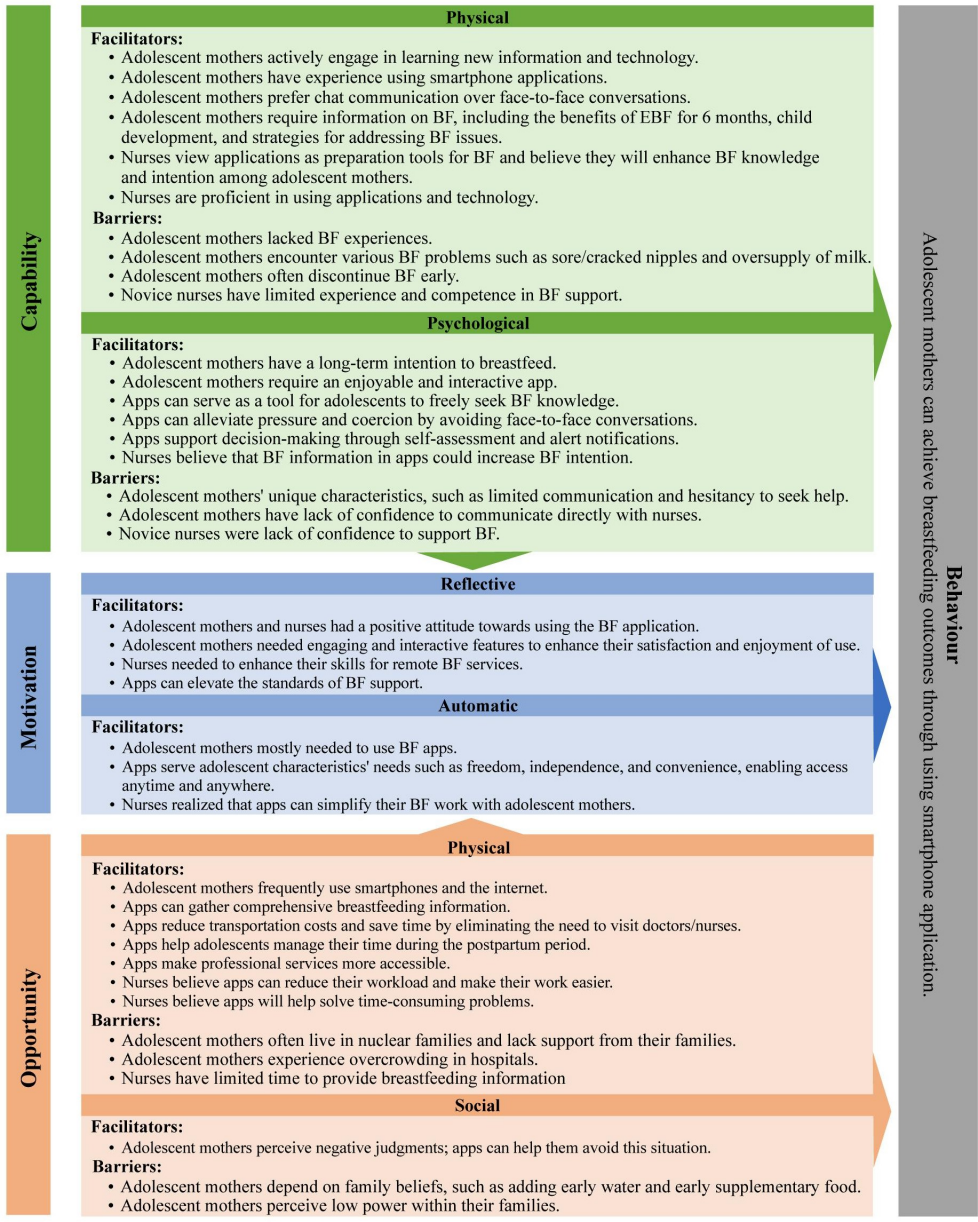
Mapping the stakeholders’ perceptions and needs affecting smartphone application design for breastfeeding support to the capability, opportunity, and motivation model of behaviour change (COM-B).

Fig 1 presents a map of the stakeholders’ perceptions and viewpoints, which affect the plan and design of a smartphone application for supporting BF practices using data obtained from the qualitative FGDs and IDIs, as well as for understanding unique adolescent developments, that is fitted to the COM-B. The defined behaviour is that all adolescent mothers are able to achieve optimal BF outcomes. To achieve this behaviour in Thailand, the smartphone application design and implementation plan should ensure that the key facilitators and barriers identified are addressed.

## Discussion

This study presented the adolescent mothers and obstetric and paediatric nurses’ perspectives on the experiences and needs of smartphone applications for BF support. We observed a positive attitude toward the use of smartphone applications as suitable technology for BF education and consultation with teenage mothers. Moreover, this health technology could address an adolescent’s problems, such as lack of support, low socioeconomic status, and social judgement. Even though most adolescents have previously used a BF smartphone application, they are still in search for the right one that could improve their satisfaction levels. The results of this study will further guide the application design and implementation plan. Many adolescents are highly skilled computer users, and they use the internet daily for different purposes [36]. More than half of these adolescents are familiar with mobile applications for BF. This technology platform gave them more autonomy, freedom, and decision-making power than dependent roles. However, the decision-making processes in adolescents are grounded in uncomplicated and emotional choices [37]. Thus, BF topics that will be designed to promote teenage self-decision-making, including correct BF position, assessment of sufficient BF, and effective breast pumping [38]. For complex BF issues, a counselling function in the application should be developed to assess and provide support from BF experts. Previous evidence found that a high level of decision-making required skills for information search and the ability to assimilate new information [39]. Moreover, a higher level of decision-making and self-efficacy is related to a high self-esteem in adolescents [40]. These relationships can simplify BF information to guide and promote adolescents’ decision-making and mental wellness.

According to adolescent development, adolescents may be concerned about their freedom during the middle stages, whereas late adolescence brings a more mature identity and the ability to make their own decisions [41]. Teenage mothers feel stress and pressure about their choices limiting their freedom to make their own decisions [11]. Furthermore, the interactive function is needed to directly consult with healthcare providers through typing of messages. This service provided the adolescent an opportunity by facilitating more accessible BF support and being a continuing-care tool due to its low cost and friendly support. According to several studies, adolescent mothers were more likely to be single parents with low educational level and family income [42, 43].

The obstetric and paediatric nurses have positive perceptions of the new BF support technology for teen mothers. In addition, nurses can have reduced workload when more teen mothers will use the smartphone application because these mothers may acquire more BF knowledge and BF intention. Regarding practical guidelines, nurses with less experience with BF support expressed that smartphone apps could create standard care. The use of mobile health application in maternity nursing care improved the mother’s knowledge, motivation, and health behaviours [44]. However, Odeh et al. [45] found that nurses identified barriers to implementing telehealth, including a lack of resources, organisational support, patient selection criteria, and technical support. Therefore, smartphone applications with valid information and support from nurses should be coordinated with the interventions to achieve optimal BF outcomes among adolescent mothers. However, nurses involved in providing BF support, particularly novice nurses, should receive extensive training.

According to a COM-B model of behavioural change, behaviour occurs as the result of interaction between three necessary conditions: capabilities, opportunities, and motivation [34]. To achieve BF outcomes, the development of BF smartphone applications needed to understand related factors based on COM-B components. Indeed, motivation to use BF apps is a core part of the model of BF behaviour, which is influenced by capabilities and opportunities to use BF apps. This study found that smartphone apps have the potential to promote and support BF practices, which require the development of necessary features such as gathering valid BF information, expert consultation, and decision-aid tools. Moreover, the capacity of apps can serve the unique characteristics of adolescent mothers. However, the service system may need to reskill and upskill BF support nurses, particularly novice nurses. According to Celik [16], the integration of BF approaches with technology could increase the initiation and continuation of BF in adolescents. Healthcare providers have significant roles to play in maintaining, protecting, and improving maternal and infant health through BF success. While opportunities are an attribute of an environment making BF behaviour, it was reported that the chance of accessing quality remote services and saving time and costs ensures adolescent equality. This service can help adolescent mothers because they are more likely to live with low SES incomes and a lack of social support [43]. However, multiple features of the application may be costly and require technology experts to collaborate. Finally, motivation is an aggregate of mental processes that generate energy and direct behaviour [34]. Adolescent mothers and nurses had a positive attitude towards using the BF application. They needed motivation, engagement, and interactive features to increase their satisfaction and enjoyment of use, which were designed for comfort and freedom. These vital components will drive BF outcomes through support for smartphone applications among adolescent mothers. An effective and comprehensive smartphone application to support BF should be developed further.

Our study has several limitations. This study collected qualitative data from one setting, which might limit the generalizability of the study findings. Moreover, the process of collecting data occurred during the coronavirus disease (COVID-19) pandemic, which might have affected the study design for data collection and perception of participants of the online platform in Zoom meeting. The FGDs were conducted via an online platform; hence, the frequent internet connection problems might have interrupted some activities and reduced the quality of data. Most participants, including both adolescent mothers and nurses, might be familiar with and have a positive attitude about healthcare technology since the study was conducted during the COVID-19 pandemic. Furthermore, adolescent mothers and nurses who were not using the real BF application might limit their experiences and perceptions. In addition, nearly half of the adolescent mothers in this study never used the BF application and participated in the same group discussion. However, they could discuss their needs and previous users’ other favourited applications to provide preferred designs and address their BF concerns. Likewise, nurses explained that their perceptions were linked to their work experiences. Regardless of these limitations, the key strengths of our study include triangulation of the results from qualitative research in FGDs and IDIs and mapping the key factors, perceptions, and needs affecting smartphone applications for BF support to the COM-B to guide the design and plan for implementing optimal BF support among adolescent mothers.

## Conclusion

The adolescent mothers and professional nurses in this study had positive attitudes toward smartphone applications for BF support. The key themes that emerged from the mothers related to the unique adolescent development and limitations of their difficult situations were as follows: need for a friendly BF tool; allow them to manage their BF activities; and enhance the accessibility and equality for BF support. The nurses perceived smartphone applications to be beneficial for their roles in providing BF support because they reduced their workload, made their work easier, improved adolescents’ BF preparations and raised BF support standards. Lastly, the COM-B displayed suitable design and implementation plan for developing BF applications in this vulnerable group. Therefore, smartphone applications to support BF should be urgently tailored to this group to encourage optimal BF outcomes as an innovation in the modern era.

## Supporting information

S1 File Focus group and interview guide. (PDF)
