# Supplementary material for: ‘The way to obtain freedom and equality’: Experiences and needs of Thai adolescent mothers in terms of the use smartphone applications for breastfeeding support
Source: PLoS One. 2024 Apr 1;19(4):e0300041. doi: 10.1371/journal.pone.0300041 (PMC10984458; doi:10.1371/journal.pone.0300041)
Supplement: S1 File — (PDF) [file pone.0300041.s001.pdf]

Focus Group Guide for Adolescent Mothers

Beginning Question

1. From your recent experiences in breastfeeding and caring for your infant in the past month, what difficulties or challenges have you encountered? How did you deal with these issues?
2. When facing difficulties, how did you solve these problems?

Leading Question

3. Which sources did you consult to address or clarify the issues you faced? Why did you choose these sources?
4. What kind of knowledge resources related to breastfeeding and infant care do you seek, and why?

Main Question

5. When discussing health-related apps, what comes to your mind and why?
6. If there were apps related to breastfeeding and infant behavior learning, what features would you like these apps to have and why?
7. What would make you choose to use an app related to breastfeeding and infant behavior learning?
8. What would encourage you to continuously use an app related to breastfeeding and infant behavior learning?
9. What would make you feel engaged and involved in using an app related to breastfeeding and infant behavior learning?
10. In your opinion, what is the most suitable time to start using an app to promote and support breastfeeding and infant behavior learning, and why?
11. How long do you think the usage duration of an app to promote and support breastfeeding and infant behavior learning should be, and why?
12. What differences do you think exist between self-education through apps and using healthcare professionals' services at hospitals to promote and support breastfeeding and infant behavior learning? How?
13. If support is provided to encourage the use of apps related to breastfeeding and infant behavior learning, what problems and obstacles do you think might arise, and why?

Additional Question

14. Do you have any additional suggestions for app developers to promote and support breastfeeding and infant behavior learning? If so, what are they?

In-depth Interview Guide for Nurses

Beginning Question

1. Have you ever used any health-related apps?
2. What are your thoughts on using health-related apps currently?

Leading Question

3. Could you share any experiences related to working with breastfeeding mothers and/or adolescent mothers?
4. What do you think about using apps to promote and support breastfeeding among adolescent mothers?
5. How do you imagine apps for promoting and supporting breastfeeding among adolescent mothers?
6. In your role as a healthcare provider, how would you like to be involved in apps for promoting and supporting breastfeeding among adolescent mothers?
7. Who do you think should be involved in ensuring the continuous and sustainable use of apps for promoting and supporting breastfeeding among adolescent mothers?
8. When do you think apps for promoting and supporting breastfeeding among adolescent mothers should be implemented, and why?
9. Who do you think should lead the utilization of apps for promoting and supporting breastfeeding among adolescent mothers, and why?
10. Do you think the implementation of apps for promoting and supporting breastfeeding among adolescent mothers affects your work? How?
11. What factors do you think influence the success of using apps for promoting and supporting breastfeeding among adolescent mothers?
12. What are the advantages and disadvantages of using apps for promoting and supporting breastfeeding among adolescent mothers?

Additional Question

13. Could you provide further recommendations for app developers to help adolescent mothers succeed in breastfeeding?
